# Intracellular lactate signaling cascade in atrial remodeling of mitral valvular patients with atrial fibrillation

**DOI:** 10.1186/1749-8090-8-34

**Published:** 2013-03-01

**Authors:** Jing Xu, Xiaohan Xu, Linjie Si, Lei Xue, Shijiang Zhang, Jianwei Qin, Yanhu Wu, Yongfeng Shao, Yijiang Chen, Xiaowei Wang

**Affiliations:** 1 Department of Thoracic and Cardiovascular Surgery, The First Affiliated Hospital of Nanjing Medical University, Nanjing, People's Republic of China

## Abstract

**Background:**

Atrial remodeling has emerged as the structural basis for the maintenance and recurrence of atrial fibrillation. Lactate signaling cascade was recently linked to some cardiovascular disorders for its regulatory functions to myocardial structural remodeling. It was hypothesized that lactate signaling cascade was involved in the maintenance and recurrence of atrial fibrillation by regulating atrial structural remodeling.

**Methods:**

Biopsies of right atrial appendage and clinical data were collected from sex- and age-matched 30 persistent atrial fibrillation, 30 paroxysmal atrial fibrillation, 30 sinus rhythm patients undergoing isolated mitral valve surgery and 10 healthy heart donors.

**Results:**

Atrial fibrillation groups had higher atrial lactate expression and this upregulated expression was positively correlated with regulatory indicators of atrial structural remodeling as reflected by severe oxidative stress injury and mitochondrial control of apoptosis.

**Conclusions:**

The present findings suggest a potential role for lactate signaling cascade in the maintenance and recurrence of atrial fibrillation and possibly represent new targets for therapeutic intervention in this disorder.

## Background

Atrial fibrillation (AF) is the most common clinically encountered arrhythmia associated with substantial cardiovascular morbidity and mortality [1]. It is present in up to 50% of patients with mitral valve disease, contributing to increased risks of systemic embolization, anticoagulant-related hemorrhage and congestive heart failure [2]. In spite of significant progress in AF treatment, the currently available therapeutic approaches have revealed major intrinsic weaknesses, including limited long-term efficacy and numerous side effects [3]. Most frequently, the notion of “upstream therapy”, targeting the processes involved in the development of the substrate of AF, has increasingly become the focus of attempts at therapeutic innovation. Successful development of “upstream therapy” depends on our ability to understand the pathophysiological mechanisms underlying AF [4].

The fundamental mechanism underlying AF is primarily characterized by electrical remodeling, but limited success rates and uncertain long-term outcomes of current therapies based on this hypothesis, indicate that apart from electrical remodeling other factors are also involved in development of AF [5]. In the recent decade, increasing experimental studies and clinical investigations have demonstrated that, in addition to electrical remodeling, atrial structural remodeling, especially inflammation, interstitial fibrosis, myocardial apoptosis and oxidative stress, occurs in maintenance and recurrence of AF, creating more favorable substrates for AF [6]. Therefore, targeting the regulation of atrial structural remodeling may be a promising therapeutic direction.

In conventional wisdom, lactate, the end product of glycolysis, is formed as the result of oxygen insufficiency and utilized under fully aerobic conditions in contracting cardiac muscle. However, rapid progress in ongoing research has shown discordant results with the prevailing view. It is now known that lactate is produced continuously under fully aerobic condition and functions as a cell signaling molecule regulation cellular redox state [7]. Under physiological conditions the heart utilizes lactate for oxygen consumption, but during pathological conditions, production of lactate increases several fold [8]. The excessive increased lactate stimulates reactive oxygen species (ROS) generation by activation several related transcription factors result in myocardial structural remodeling including oxidative stress and mitochondrial control of apoptosis, which named as lactate signaling cascade [9]. Significant association was identified between lactate signaling cascade and cardiovascular diseases such as ischemic-reperfusion injury [10] and volume overload [11] or myocardial infarction [12] induced heart failure.

Therefore, we hypothesized that the lactate signaling cascade was involved in development and perpetuation of AF by regulating atrial structural remodeling. To test this hypothesis, in the present study we measured lactate signaling cascade in right atrial appendage (RAA) tissues of AF and sinus rhythm (SR) patients matched with normal control (NC), and the positive results may be useful in designing targets for future upstream therapy.

## Methods

### Patients

We have built a biobank for AF research in the First Affiliated Hospital of Nanjing Medical University since November 2007 [13,14]. The present study was conducted according to the Helsinki Declaration and approved by the ethics committees of Nanjing Medical University. Only if a patient gave written informed consent, we could collect his atrial tissue during surgery and detail clinical data.

In the present study, we included the patients undergoing isolated mitral valve surgery and excluded some of them as follows: 1) patients who exceeded 65 years old or had a history of cancer; 2) patients with concomitant coronary artery disease; 3) patients with aortic or valvular calcification; 4) patients who suffered bacterial endocarditis or had detected rheumatic activity; 5) patients with complicated diabetes or renal dysfunction (serum creatinine>136 μmol/L). Finally, we recruited 30 patients with preoperative paroxysmal atrial fibrillation (PaAF) and 30 sex- and age-matched patients with persistent atrial fibrillation (PeAF) and SR. NC were 10 sex- and age-matched healthy heart donors. They were trauma victims and were free of cardiovascular pathology and documented AF.

### Echocardiographic evaluation

Before surgery, each patient received comprehensive transthoracic echocardiographic examination (GE VIVID5, General Electric Medical, Wisconsin, USA). As for the recommendation of the American Society of Echocardiography, the left ventricle dimension was evaluated using left ventricular end-diastolic and end-systolic diameters. Left ventricular ejection fraction was assessed by means of M-mode echocardiography (Teichholtz method) and atrial dimension was measured in the parasternal long axis view at endsystole. Pulmonary artery systolic pressure was derived from the regurgitant jet of tricuspid regurgitation using systolic transtricuspid pressure gradient calculated by the modified Bernoulli equation (ΔP=4v2, where v is maximal tricuspid regurgitant jet velocity in m/s) and the addition of 10 mm Hg for right atrial pressure as previously performed. All measurements were performed by 2 observers.

### Tissue collection

RAA tissue of each patient was obtained from the cannulation site before starting extracorporeal circulation. Sampling site was the same because of surgical maneuver. RAA specimens were obtained from donor hearts before perfusion.

### Mitochondrial isolation

Fresh RAA tissue was immediately washed with prechilled isolation buffer containing 220 mM mannitol, 70 mM sucrose, and 5 mM MOPS, pH 7.4. The RAA tissue was then minced and homogenized by a motor-driven Teflon pestle in the isolation buffer. The homogenate was centrifuged at 500 g for 10 min at 4°C, and supernatant was collected by passing it through cheesecloth. The filtrate was centrifuged at 5,000 g for 10 min at 4°C. The pellet was washed again with isolation buffer and gently resuspended in respiration buffer (225 mM mannitol, 70 mM sucrose, 10 mM KH2PO4, and 1 mM EGTA, pH 7.2). Protein concentration of the mitochondria preparation was determined by the Lowry method.

### Lactate and ROS measurements

Atrial lactate expression was determined according to the method [15] that depends on oxidation of lactate by lactate dehydrogenase in the presence of nicotinamide adenine dinucleotide (NAD^+^). The formed NADH was measured at 340 nm. Results were expressed as μmol/g wet tissue. Atrial lipid peroxidation products were estimated by determination of the thiobarbituric acid reactive substances (TBARS) [16] that were measured as malondialdehyde and expressed as ng/mg wet tissue.

### Western blot

Frozen RAA or cardiac mitochondria were used for protein isolation as described previously [17]. Briefly, the proteins were separated by SDS-PAGE and transferred onto polyvinylidene difluoride membranes (Amersham). The membranes were incubated with the appropriate primary antibodies anti-cytochrome-c, anti-cleaved-caspase9, anti-cleaved-caspase3, anti-COx-IV (Cell Signaling Technology), anti-Monocarboxylate transporter 1 (MCT1) (Chemicon International), GAPDH (Santa Cruz) followed by incubation with peroxidase-conjugated secondary antibodies. The signals were detected with the ECL system (Pierce). The same membranes were probed with anti-beta-actin (Sigma) after being washed with stripping buffer. The signals were quantified by scanning densitometry with the Image J analysis system. The results from each experimental group were expressed as relative integrated intensity compared with the control group measured at the same time.

### Statistical analysis

For the comparison among groups, one-way ANOVA test (normally distributed) or Mann–Whitney test (2 groups, non-normally distributed) and Kruskal-Wallis test (n groups, non-normally distributed) was used for continuous variables, and *χ*^2^ test was utilized for categorical variables. The statistical analysis was performed with the GBSTAT statistical analysis package (version 9.0, Dynamic Microsystems, Inc).

## Results

### Clinical characteristics

The characteristics of the SR, PaAF, PeAF and NC groups included in the analysis are summarized in Table 1. There were no statistically significant differences between groups in terms of age and sex. C-reactive protein and pulmonary artery systolic pressure, as well as preoperative length of stay were higher or longer in two AF groups than SR group. The history of valvular disease was longer in PeAF than that of SR and PaAF. Left and right atrial diameters were largest in PeAF, followed by PaAF and SR. The proportion of rheumatic cause and left atrial thrombus was higher in PeAF than SR, and the former was also higher in PeAF than PaAF.

**Table 1:** Clinical characteristics of study population

**Parameters**	**SR**	**PaAF**	**PeAF**
**Sex, M/F (n)**	**15/15**	**15/15**	**15/15**
**Age (years)**	**48.4±11.2**	**49.1.±10.1**	**48.7±10.8**
**Body mass index (kg/m**^**2**^**)**	**23.2±2.8**	**22.9±2.4**	**22.2±3.1**
**Heart rate (beats/min)**	**74±9**	**77±11**	**78±15**
**Mean artery pressure (mm Hg)**	**84.6±7.2**	**86.6±9.4**	**83.2±10.2**
**C-reactive protein (mg/L)**	**2.86±1.02**	4.32±0.94*	5.04±1.21*^,^**
**Duration of valvular disease (years)**	**4.9±4.8**	**6.9±5.7**	12.5±7.2*^,^**
**NYHA class I/II/III/IV (n)**	**4/9/14/3**	**3/7/15/5**	**2/4/17/7**
**Echocardiographic parameters**			
**LVDd (mm)**	**48.6±7.4**	**50.4±6.8**	**49.2±8.1**
**LVDs (mm)**	**31.8±4.7**	**32.3±5.2**	**31.1±4.9**
**EF (%)**	**63.6±3.9**	**62.8±4.4**	**63.4±4.1**
**LAD (mm)**	**41.7±6.4**	52.4±8.2*	59.9±9.4*^,^**
**RAD (mm)**	**37.7±4.1**	42.5±4.6*	46.3±5.7*^,^**
**PASP (mm Hg)**	**34.2±7.7**	44.1±11.3*	51.2±14.9*^,^**
**Left atrial thrombus (n)**	**0**	**3**	8*^,^**
**Preoperative length of stay (days)**	**9.6±4.2**	12.2±5.6*	15.8±6.8*^,^**
**Cause of mitral valve disease (n)**			
**Rheumatic/degenerative**	**22/8**	**24/6**	28/2*^,^**
**Preoperative drug (n)**			
**Digitalis**	**30**	**30**	**30**
**Calcium-channel blocker**	**7**	**5**	**8**
**Beta-blocker**	**3**	**4**	**6**
**ACE-I**	**5**	**7**	**10**

Values are presented as mean±SD or number of patients.

*ACE*-*I *angiotensin-converting enzyme inhibitor, *EF* ejection fraction, *LAD *left atrial diameter, *LVDd *left ventricular end-diastolic diameter, *LVDs *left ventricular end-systolic diameter, *NYHA* New York Heart Association, *PASP *pulmonary artery systolic pressure, *RAD *right atrial diameter.

* p<0.05, reference group is the SR group.

** *p*<0.05, reference group is the PaAF group.

### Biochemical parameters

**Figure 1:**
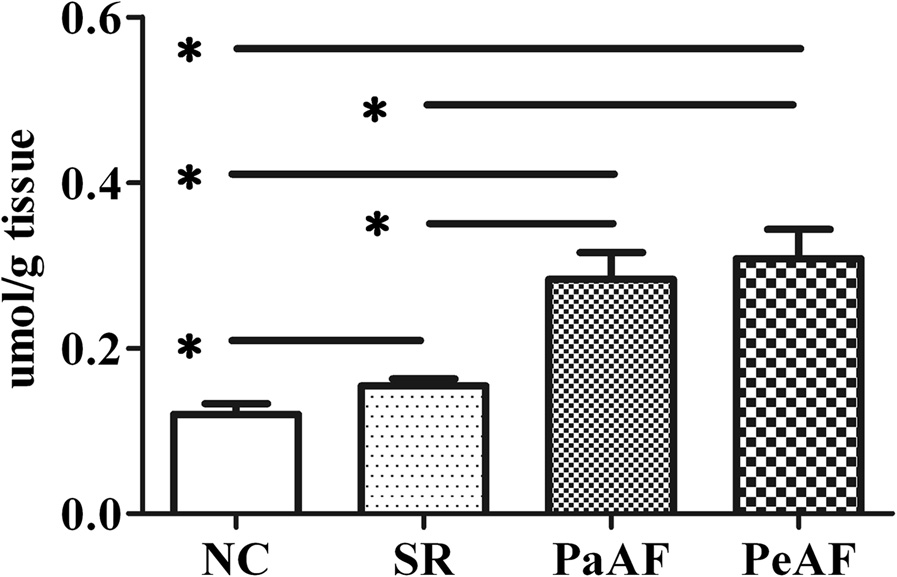
The expression of lactate in RAA tissues.

**Figure 2:**
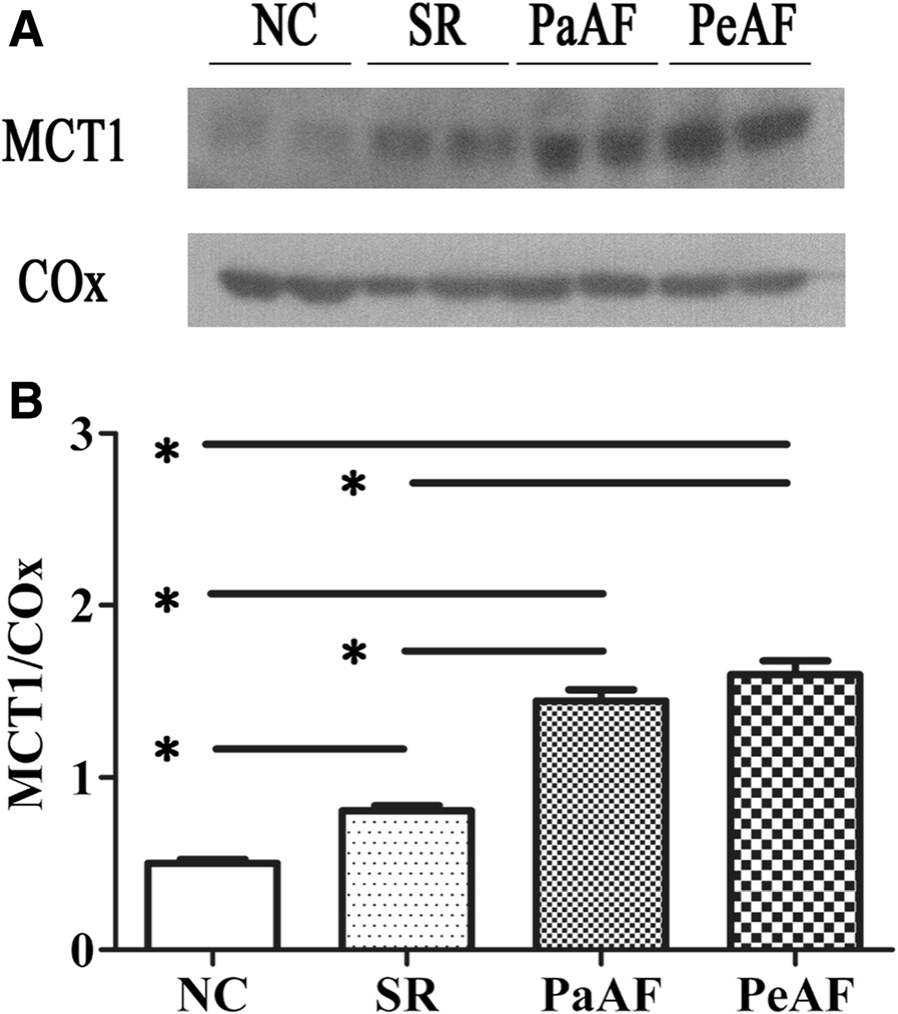
The protein expression of MCT1 in mitochondrial membrane. (A) Representative MCT1 and COx immunoblotting were shown in NC, SR, PaAF and PeAF groups (B) The different levels of MCT1/ Cox were determined among above-mentioned groups.

The lactate expression of RAA tissues were increased gradient in SR, PaAF and PeAF, elevated 1.3, 2.3 and 2.6 folds respectively compared with NC, however, no significant difference was found between PaAF and PeAF (*P*=0.61) (Figure 1). Similarly, in comparison with NC, the TBARS content in SR, PaAF and PeAF increased 1.9, 5.5, 6.6 folds respectively and between PaAF and PeAF there was no significant difference (*P*=0.23) (Figure 2).

### Expression of lactate signaling cascade in RAAs

**Figure 3:**
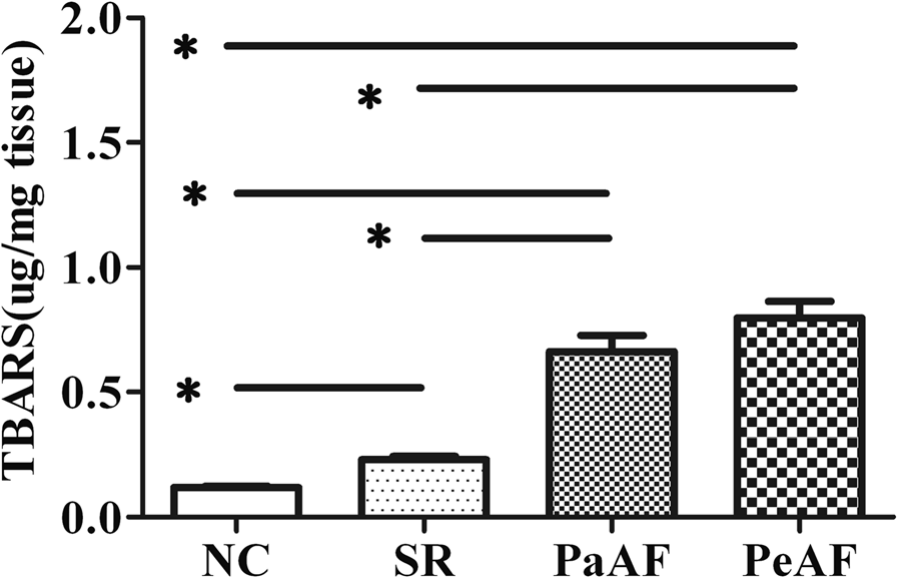
The level of ROS in RAA tissues by TBARS.

**Figure 4:**
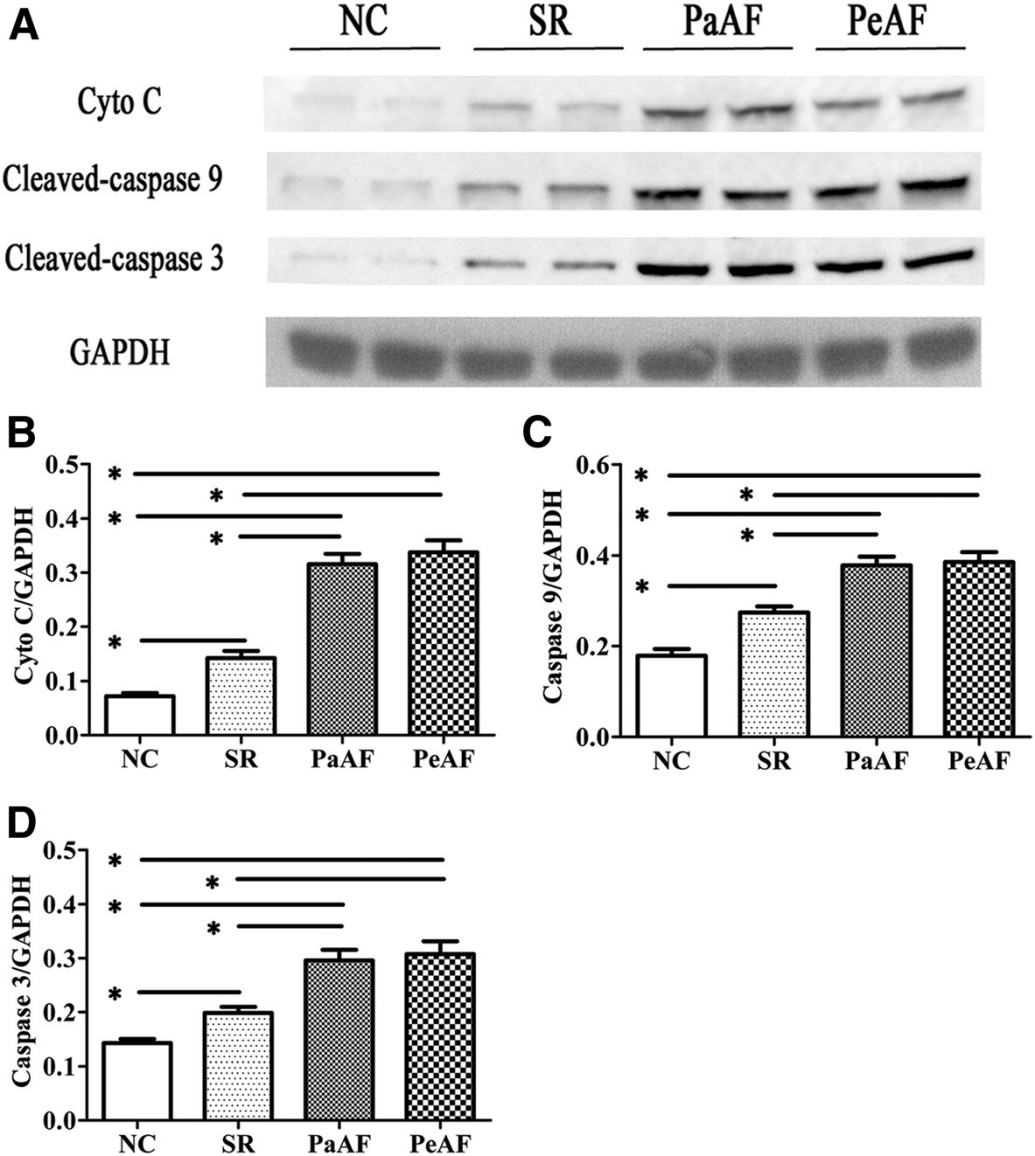
The proteins expression of mitochondrial apoptosis pathway in RAA tissues. (A) Representative cyto c, cleaved-caspase 9, cleaved-caspase 3 and GAPDH immunoblotting were shown in NC, SR, PaAF and PeAF groups. The different levels of cyto c/GAPDH (B), cleaved-caspase 9/GAPDH (C), cleaved-caspase 3/GAPDH (D) were determined respectively among above-mentioned groups.

Western blot analysis (Figure 3) prompted that the expression of MCT1 in PaAF and PeAF was significantly higher compared with that in SR and NC, although there was no difference between PaAF and PeAF (*p*=0.17). Expressions of cytochrome c (Figure 4B), cleaved-caspase9 (Figure 4C) and cleaved-caspase3 (Figure 4C) were found highest in PaAF and PeAF, followed by SR and NC. However, no significant difference was identified between PaAF and PeAF (*p*=0.17, *p*=0.81, *p*=0.72 for cytochrome c, cleaved-caspase9 and cleaved-caspase3 respectively).

## Discussion

**Figure 5:**
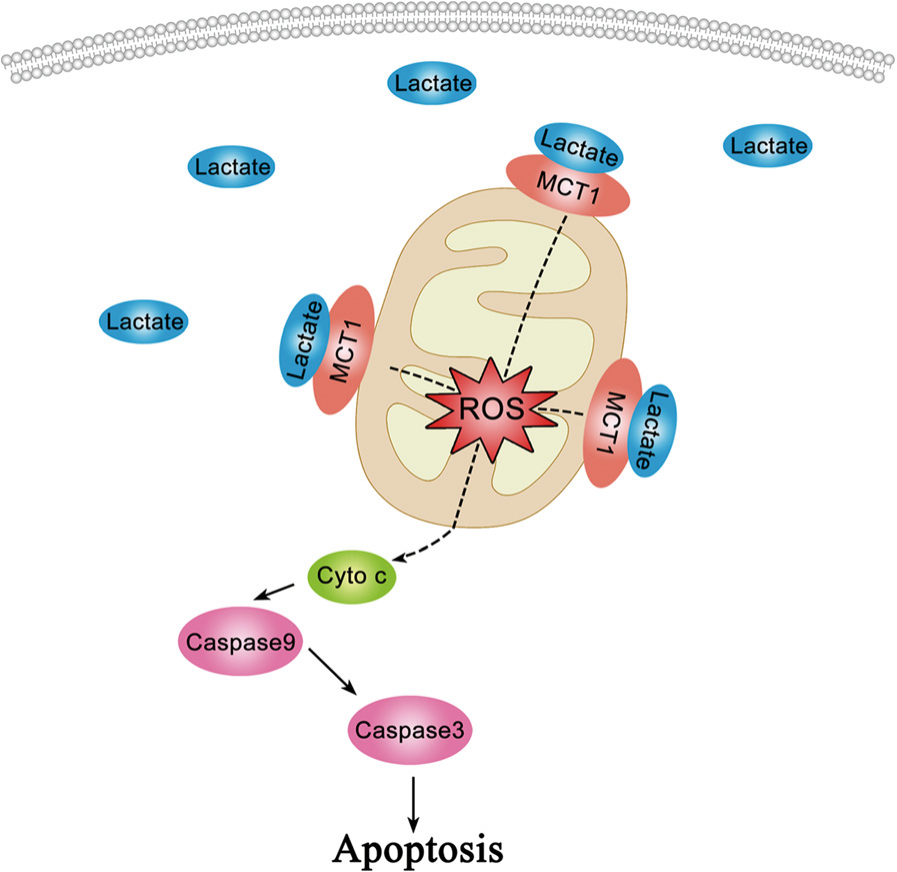
The simplified scheme of interactions within lactate signalling cascade.

To our knowledge, this is the first study to provide evidence that lactate signaling cascade may play a role in maintenance and recurrence of AF. An increasing gradient of protein expression of lactate as well as MCT1 were identified in NC, SR and AF groups. Furthermore, the redox level and mitochondrial apoptosis proteins were also increased consistent with lactate expression in RAAs. Therefore, the present data support that the lactate signaling cascade might be one of the regulatory systems in the atrial structural remodeling of AF Figure 5.

As described previously, intracellular lactate shuttle hypothesis posits that in normal physiological circumstances intracellular lactate is produced as the result of glycolysis and glycogenolysis in the cytosol and balanced by oxidation in mitochondria. The presence of MCT1 in myocardial mitochondrial membrane allows mitochondrial lactate influx and oxidation. However, during pathological conditions such as hypoxia or ischemia the myocardial energy demands increases, glucose uptake and glycogenolysis raise many times which causes lactate concentration elevation. The corresponding increased expression of MCT1 would likely help the cell to attenuate intracellular acidification [18]. In experimental animal model, two previous studies have identified convincingly positive correlation between the dysfunction of lactate metabolism and cardiovascular disorders. In chronic volume overload model, myocardial hypertrophy and subsequent congestive heart failure engendered a greater maximal lactate transport capacity across the cardiac myocytes sarcolemma along with an increased in MCT1 protein content [11]. Moreover, in another myocardial infarction model, similar maximum lactate influx into isolated cardiomyocytes and increased MCT1 protein level were also observed [12]. These findings reveal that excessive accumulation of lactate and high expression of MCT1 exists in cardiovascular diseases. In accordance with the previous findings, the present study has explored for the first time the expression of lactate were significantly increased in AF groups compared with NC, SR groups, and that the increased lactate expression could further promoted the protein expression of MCT1. Therefore, together with the previous findings, it is not inconceivable that enhanced lactate expression is not only as an epiphenomenon, but may also have an independent effect on the development and progression of the diseases.

Fortunately, two noteworthy observations preliminary unveil the veil. First the L6 cells incubated with exogenous lactate rapidly activated ROS production, upregulated the total mitochondrial mass and increased mitochondrial electron transport in vitro, indicating that lactate signaling affects ROS production and mitochondrial biological activities [19]. Second, cytosolic acidification induced by lactate in CHO cells was given rise to mitochondrial control of apoptosis including mitochondrial dysfunction, cytochrome c release, and an increased in caspase-3 enzymatic activity. However, the anti-lactate treatment could completely reverse the effects [20]. Convincingly, these findings suggest that in addition to serving as an oxidizable substrate and gluconeogenic precursor, lactate also is an important cell-signaling molecule involved in regulation of cell redox state and mitochondrial biogenesis.

To illustrate lactate signaling in the cardiac myocyte, Kubasiak et al. [21] reported that hypoxia did not induce cardiac myocyte apoptosis in the absence of lactate acidosis and this procedure was mediated by BNIP3, a member of the Bcl-2 family of mitochondrial apoptosis-regulating proteins. Subsequently, a more in-depth study investigated the functional role of lactate signaling both *in vivo* and in vitro [22]. In hearts of mice subjected to global ischemia the expression of lactate transport protein MCT1 was increased at the start of reperfusion. Blocking the function of MCT1 by its competitive inhibitor, the left ventricular performance was worse and the infarct size was increased. In vitro the MCT1 inhibitor increased the death of HL-1 cells stimulated with hydrogen peroxide as well. Therefore, it must be mentioned that disruption of lactate metabolism can lead to activate lactate signal cascade, then contribute to aggravate ROS release and myocyte apoptosis, which is in line with the previous findings in L6 and CHO cells. In our study we observed that a significant increasing ROS level was identify in AF compared with NC, SR groups. Besides the expression of mitochondrial apoptosis pathway proteins cytochrome c, cleaved-caspase9, cleaved-caspase3 was also found higher in AF than NC, SR groups. These data suggest that during AF, atrial myocytes might more dependent on glycolysis for their ATP production and thus require transport more lactate to mitochondrial oxidation by upregulation MCT1. Although the increased expression of intracellular lactate shuttles transport protein MCT1 facilitates lactate oxidation in atrial myocytes from cytosol to mitochondria, lactic acidosis is still inevitable happened, resulting in atrial remodeling consist of oxidative stress damage and mitochondrial control of apoptosis.

### Study limitations

First in our hospital-based study, the sample size was moderate, and therefore further studies with larger sample size are warranted to confirm the findings. Second, since the majority of our patients suffered from rheumatic mitral valve disorders, local inflammatory response may influence myocardial metabolism and atrial remodeling. Accordingly, to attenuate this effect, patients with detected rheumatic activity were excluded in the present study. Finally, Due to the nature of our study, whether lactate signaling cascade changes represent a cause-effect relationship or solely an association remains uncertain. However, activation of the lactate signaling cascade may not only be a marker but also a mediator in the development and perpetuation of AF, at least partly, by promoting atrial remodeling. Future cell and animal studies should address this issue.

## Conclusions

The present study revealed that atrial lactate expression increased in AF atrial myocytes, and was associated with the presence of oxidative stress damage and cardiomyocytes apoptosis, suggesting that the cascade may contribute to the development and progression of AF by regulating atrial remodeling. Thus, the current data indicates a potential role for known mediators of lactate signaling cascade in the pathogenesis of AF and possibly represents new targets for therapeutic intervention in AF.

## Abbreviations

AF: Atrial fibrillation; PeAF: Persistent AF; PaAF: Paroxysmal AF; SR: Sinus rhythm; NC: Normal controls; ROS: Reactive oxygen species; RAA: Right atrial appendage; TBARS: Thiobarbituric acid reactive substances; MCT: Monocarboxylate transporter

## Competing interests

We declare that we have no competing interests.

## Authors’ contributions

JX, XHX and XWW participated in the design of the study and coordination, YFS, JWQ, YHW and SJZ participated in the samples and data collection, LJS and LX performed the statistical analysis, YJC helped to draft and modified the manuscript. All authors read and approved the final manuscript.

## References

[B1] Nattel S Opie LH Controversies in atrial fibrillation Lancet 2006 367 262 272 10.1016/S0140-6736(06)68037-9 16427496

[B2] Chen MC Chang JP Chen YL Surgical treatment of atrial fibrillation with concomitant mitral valve disease: an Asian review Chang Gung Med J 2008 31 538 545 19241892

[B3] Nattel S Carlsson L Innovative approaches to anti-arrhythmic drug therapy Nat Rev Drug Discov 2006 5 1034 1049 10.1038/nrd2112 17139288

[B4] Calò L Martino A Sciarra L Ciccaglioni A De Ruvo E De Luca L Sette A Giunta G Lioy E Fedele F Upstream effect for atrial fibrillation: still a dilemma? Pacing Clin Electrophysiol 2011 34 111 128 10.1111/j.1540-8159.2010.02942.x 21029134

[B5] Chang D Zhang S Yang D Gao L Lin Y Chu Z Jiang X Yin X Zheng Z Wei X You D Xiao X Cong P Bian X Xia Y Yang Y Effect of epicardial fat pad ablation on acute atrial electrical remodeling and inducibility of atrial fibrillation Circ J 2010 74 885 894 10.1253/circj.CJ-09-0967 20379001

[B6] McManus DD Shaikh AY Abhishek F Vasan RS Atrial fibrillation and heart failure parallels: lessons for atrial fibrillation prevention Crit Pathw Cardiol 2011 10 46 51 10.1097/HPC.0b013e31820e1a4b 21562376 PMC3220939

[B7] Gladden LB Lactate metabolism: a new paradigm for the third millennium J Physiol 2004 558 5 30 10.1113/jphysiol.2003.058701 15131240 PMC1664920

[B8] Bonen A The expression of lactate transporters (MCT1 and MCT4) in heart and muscle Eur J Appl Physiol 2001 86 6 11 10.1007/s004210100516 11820324

[B9] Hashimoto T Brooks GA Mitochondrial lactate oxidation complex and an adaptive role for lactate production Med Sci Sports Exerc 2008 40 486 494 10.1249/MSS.0b013e31815fcb04 18379211

[B10] Halestrap AP Wang X Poole RC Jackson VN Price NT Lactate transport in heart in relation to myocardial ischemia Am J Cardiol 1997 80 17A 25A 10.1016/S0002-9149(97)00454-2 9293952

[B11] Evans RK Schwartz DD Gladden LB Effect of myocardial volume overload and heart failure on lactate transport into isolated cardiac myocytes J Appl Physiol 2003 94 1169 1176 12571142 10.1152/japplphysiol.00778.2002

[B12] Jóhannsson E Lunde PK Heddle C Sjaastad I Thomas MJ Bergersen L Halestrap AP Blackstad TW Ottersen OP Sejersted OM Upregulation of the cardiac monocarboxylate transporter MCT1 in a rat model of congestive heart failure Circulation 2001 104 729 734 10.1161/hc3201.092286 11489783

[B13] Cao H Wang J Xi L Røe OD Chen Y Wang D Dysregulated atrial gene expression of osteoprotegerin/receptor activator of nuclear factor-κB (RANK)/RANK ligand axis in the development and progression of atrial fibrillation Circ J 2011 75 2781 2788 10.1253/circj.CJ-11-0795 22001292

[B14] Cao H Li Q Li M Od R Wu Z Zhou Q Cao B Chen B Chen Y Wang D Osteoprotegerin/RANK/RANKL axis and atrial remodeling in mitral valvular patients with atrial fibrillation Int J Cardiol 2011 Epub ahead of print 10.1016/j.ijcard.2011.11.099 22178057

[B15] Ahmed LA Salem HA Mawsouf MN Attia AS Agha AM Cardioprotective effects of ozone oxidative preconditioning in an *in vivo* model of ischemia/reperfusion injury in rats Scand J Clin Lab Invest 2012 72 345 354 10.3109/00365513.2012.663100 22862559

[B16] Hermes-Lima M Willmore WG Storey KB Quantification of lipid peroxidation in tissue extracts based on Fe(III)xylenol orange complex formation Free Rad Biol Med 1995 19 271 280 10.1016/0891-5849(95)00020-X 7557541

[B17] Brooks GA Brown MA Butz CE Sicurello JP Dubouchaud H Cardiac and skeletal muscle mitochondria have a monocarboxylate transporter MCT1 J Appl Physiol 1999 87 1713 1718 10562613 10.1152/jappl.1999.87.5.1713

[B18] Halestrap AP Wilson MC The monocarboxylate transporter family-role and regulation IUBMB Life 2012 64 109 119 10.1002/iub.572 22162139

[B19] Hashimoto T Hussien R Oommen S Gohil K Brooks GA Lactate sensitive transcription factor network in L6 cells: activation of MCT1 and mitochondrial biogenesis FASEB J 2007 21 2602 2612 10.1096/fj.07-8174com 17395833

[B20] Jeong D Kim TS Lee JW Kim KT Kim HJ Kim IH Kim IY Blocking of acidosis-mediated apoptosis by a reduction of lactate dehydrogenase activity through antisense mRNA expression Biochem Biophys Res Commun 2001 289 1141 1149 10.1006/bbrc.2001.6091 11741311

[B21] Kubasiak LA Hernandez OM Bishopric NH Webster KA Hypoxia and acidosis activate cardiac myocyte death through the Bcl-2 family protein BNIP3 Proc Natl Acad Sci USA 2002 99 12825 12830 10.1073/pnas.202474099 12226479 PMC130544

[B22] Martinov V Rizvi SM Weiseth SA Sagave J Bergersen LH Valen G Increased expression of monocarboxylate transporter 1 after acute ischemia of isolated, perfused mouse hearts Life Sci 2009 85 379 385 10.1016/j.lfs.2009.07.001 19604494

